# Diffusion-weighted imaging complements T2-weighted MRI for tumour response assessment in squamous anal carcinoma

**DOI:** 10.1007/s00330-023-09942-0

**Published:** 2023-07-18

**Authors:** Davide Prezzi, Keerthini Muthuswamy, Ashik Amlani, Kasia Owczarczyk, Ahmed Elowaidy, Tina Mistry, Paul Bassett, Vicky Goh

**Affiliations:** 1 School of Biomedical Engineering and Imaging Sciences, King’s College London, King’s Health Partners, London, UK; 2 Department of Radiology, Guy’s and St Thomas’ NHS Foundation Trust, London, UK; 3 Statsconsultancy Ltd., Amersham, UK

**Keywords:** Magnetic resonance imaging, Diffusion magnetic resonance imaging, Anus neoplasms, Carcinoma, squamous cell, Chemoradiotherapy

## Abstract

**Objectives:**

A published tumour regression grade (TRG) score for squamous anal carcinoma treated with definitive chemoradiotherapy based on T2-weighted MRI yields a high proportion of indeterminate responses (TRG-3). We investigate whether the addition of diffusion-weighted imaging (DWI) improves tumour response assessment in the early post treatment period.

**Materials and methods:**

This retrospective observational study included squamous anal carcinoma patients undergoing MRI before and within 3 months of completing chemoradiotherapy from 2009 to 2020. Four independent radiologists (1–20 years’ experience) scored MRI studies using a 5-point TRG system (1 = complete response; 5 = no response) based on T2-weighted sequences alone, and then after a 12-week washout period, using a 5-point DWI-TRG system based on T2-weighted and DWI. Scoring confidence was recorded on a 5-point scale (1 = low; 5 = high) for each reading and compared using the Wilcoxon test. Indeterminate scores (TRG-3) from each reading session were compared using the McNemar test. Interobserver agreement was assessed using kappa statistics.

**Results:**

Eighty-five patients were included (mean age, 59 years ± 12 [SD]; 55 women). T2-weighted TRG-3 scores from all readers combined halved from 24% (82/340) to 12% (41/340) with DWI (*p* < 0.001). TRG-3 scores changed most frequently (41%, 34/82) to DWI-TRG-2 (excellent response). Complete tumour response was recorded clinically in 77/85 patients (91%). Scoring confidence increased using DWI (*p* < 0.001), with scores of 4 or 5 in 84% (287/340). Interobserver agreement remained fair to moderate (kappa range, 0.28–0.58).

**Conclusion:**

DWI complements T2-weighted MRI by reducing the number of indeterminate tumour responses (TRG-3). DWI increases radiologist’s scoring confidence.

**Clinical relevance statement:**

Diffusion-weighted imaging improves T2-weighted tumour response assessment in squamous anal cancer, halving the number of indeterminate responses in the early post treatment period, and increases radiologists’ confidence.

**Key Points:**

- *Tumour response based on T2-weighted MRI is often indeterminate in squamous anal carcinoma.*
- *Diffusion-weighted imaging alongside T2-weighted MRI halved indeterminate tumour regression grade scores assigned by four radiologists from 24 to 12%.*
- *Scoring confidence of expert and non-expert radiologists increased with the inclusion of diffusion-weighted imaging.*

**Supplementary information:**

The online version contains supplementary material available at 10.1007/s00330-023-09942-0.

## Introduction

Squamous carcinoma of the anal canal is on the rise worldwide, with an annual incidence of 0.5–2 in 100,000 [1]. Definitive radiotherapy with concomitant mitomycin C and 5-fluorouracil (or capecitabine) is the therapy of choice for localised disease, with good outcomes [2, 3]. Timely identification of locoregional treatment failure, occurring in a minority of cases (11–14%) [4, 5], allows these patients to be considered for salvage surgery, which in turn leads to local pelvic control in approximately 60% of cases and to a 5-year survival rate of 30–60% [2]. Early detection of salvageable local disease relapse during imaging response assessment and surveillance is key.

MRI is recommended for locoregional staging and response assessment [2, 3, 6, 7], and has a growing role in radiotherapy planning [8]. High-resolution T2-weighted sequences are typically obtained in planes parallel and perpendicular to the anal canal. MRI tumour response assessment based on T2-weighted sequences can be challenging in the early post treatment period, due to the overlapping features between therapy-induced inflammation (hyperintense tissue oedema mixed with hypointense fibrosis, anatomical distortion) and residual tumour, typically intermediate in signal [9]. A 5-point MRI tumour regression grade (TRG) system based on T2-weighted sequences has been utilised to classify squamous anal carcinoma tumour response to chemoradiotherapy [10]. In a prospective single-centre cohort, the number of indeterminate TRG scores (TRG-3) was considerable in the early post treatment period, corresponding to 58% of the total at 3 months and to 26% of the total at 6 months post chemoradiotherapy, emphasising the problem posed by treatment-related inflammation.

Diffusion-weighted imaging (DWI) is used routinely to aid the assessment of a variety of abdominal malignancies [11, 12]. Specifically, in rectal cancer treated with neoadjuvant chemoradiotherapy, DWI increases diagnostic accuracy in the evaluation of complete response [13] and detection of small-volume residual tumour before endoscopy [14]. We hypothesised that DWI could benefit early tumour response evaluation in squamous anal carcinoma and improve the diagnostic confidence of non-expert radiologists. Thus, the primary aim of this study was to determine whether DWI improves tumour response assessment by reducing indeterminate responses (TRG-3) in the early post treatment period. Secondary aims were to assess the impact of DWI on subjective TRG scoring confidence and interobserver agreement amongst expert and non-expert radiologists.

## Materials and methods

### Patients

**Fig. 1:**
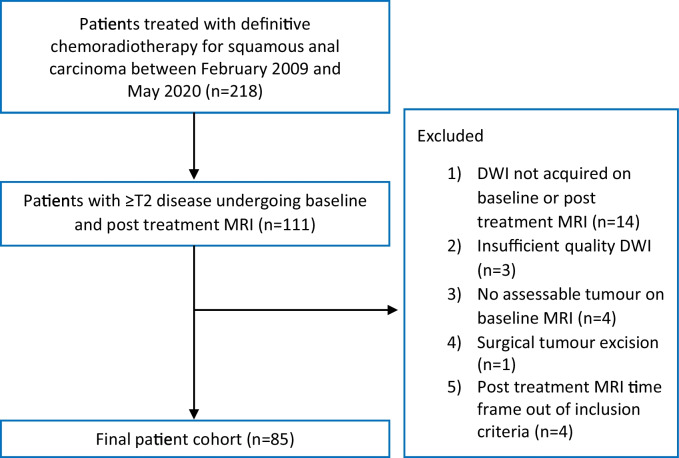
Patient flowchart

Institutional board waiver of informed consent was obtained for this retrospective study of consecutive MRI data obtained as part of the standard care pathway. Patients with biopsy-proven squamous cell carcinoma of the anal canal undergoing treatment with definitive chemoradiotherapy between February 2009 and May 2020 were identified from the Picture Archiving and Communication System (PACS) and electronic patient record (EPR) of a tertiary care institution (Guy’s and St Thomas’ NHS Foundation Trust). Inclusion criteria were baseline and post treatment MRI (within 3 months of treatment completion) available from PACS; TNM 8th ed. T2 stage; or greater tumours [15], equivalent to tumour diameter >2 cm. Exclusion criteria were absence of DWI on baseline or post treatment MRI; DWI of insufficient diagnostic quality; no visible tumour on baseline MRI; and prior tumour surgical excision. The patient flowchart is shown in Fig. 1.

### Treatment and clinical follow-up

Radiotherapy was delivered to a mean dose of 50.86 Gy (range 50.4–54 Gy) using a linear accelerator (Elekta or Varian) applying a 3D conformal or intensity-modulated technique. Concomitant chemotherapy consisted of mitomycin C (12 mg/m^2^ on day 1) with either 5-fluorouracil (1000 mg/m^2^/day, continuous venous infusion, on days 1–4 and 29–32) or capecitabine orally (825 mg/m^2^ twice a day on radiation days).

Following completion of chemoradiotherapy, patients were clinically assessed at 8–10 weeks, then every 3 months for the first 2 years, and every 6 months afterwards as per standard institutional practice. Endoscopic evaluation ± MRI/CT was undertaken if canal ± locoregional/distant recurrence was suspected clinically.

### MRI acquisition

Patients were scanned supine on one of five 1.5- or 3.0-Tesla MRI scanners (Magnetom Avanto, Aera or Skyra, Siemens Healthineers) using an 18-channel pelvic phased array coil. The examination protocol included axial and sagittal T2-weighted turbo spin-echo (TSE) sequences covering the whole pelvis, and high-resolution small-field-of-view T2-weighted TSE sequences perpendicular and parallel to the anal canal. DWI consisted of a single-shot spin-echo echo planar imaging (EPI) axial diffusion-weighted sequence encompassing the pelvis with three *b*-values (0, 100, 800 s/mm^2^). Apparent diffusion coefficient (ADC_0-800_) maps and calculated high *b*-value images (*b* = 1400 or 1600 s/mm^2^) were created automatically at the time of acquisition. Patients did not undergo any additional preparation prior to the examination. Typical acquisition parameters are summarised in Supplemental Table 1.

### Image analysis

Baseline and post treatment MRI were evaluated sequentially by four independent observers, blinded to clinical outcome: two senior radiology residents (K.M., A.A.), with 1 year’s experience in oncologic pelvic MRI but no specific experience in staging squamous anal carcinoma (referred to as ‘non-expert observers’) and two subspecialist radiologists (D.P., V.G.) with over 10 years’ experience (‘expert observers’).

Anonymised scans were presented in a randomised order. Post treatment MRIs were assessed next to baseline MRIs on dual monitors (Sectra IDS7 workstations, Sectra AB). The first reading session was based on multiplanar T2-weighted sequences alone. Tumour response was evaluated according to a previously published 5-point tumour regression grade (TRG) score [10], outlined in Table 1. In addition to TRG, the following primary tumour characteristics were recorded: size (maximum diameter in any plane); location (lower canal, mid canal, upper canal/anorectum); invasion of adjacent structures (prostate, vagina, ischioanal fossa). The second reading session, separated from the first by a 12-week wash out period, included T2-weighted and DWI assessed in conjunction. Multiplanar T2 sequences, acquired/calculated *b*-value images and corresponding ADC maps were displayed simultaneously. DWI image quality was scored subjectively as inadequate, adequate or good by each observer, documenting the nature of image degradation as free text, when present. Tumour response was re-scored according to a modified DWI-TRG system, outlined in Table 1. Post treatment DWI images were regarded positive for residual disease when diffusion restriction (signal hyperintensity on high *b*-value images matched by hypointensity on ADC map) remained present at the site of the tumour, excluding linear diffusion restriction spatially matched to anorectal mucosa. A single-slice, free-hand region of interest was drawn around the tumour on ADC maps, with reference to the corresponding DWI and T2-weighted images, and the mean tumour ADC value recorded for baseline MRI. During both reading sessions, observers scored their subjective confidence in assessing tumour response on a scale of 1 (low) to 5 (high).

**Table 1:** Summary of tumour regression grade (TRG) systems applied

Grade	Description
A. Published TRG system based on T2-weighted MRI only	
1	Complete response with no evidence of tumour and normal appearances of the anus
2	Excellent response with only low signal post treatment fibrotic change and no evidence of tumour
3	Moderate response with indeterminate heterogeneous signal intensity at the tumour site
4	Minimal response with reduction in size but evidence of intermediate tumour signal in keeping with residual disease
5	No response of the primary tumour or frank tumour progression
B. Proposed DWI-TRG system based on T2-weighted plus diffusion-weighted MRI	
1	Complete response with no evidence of tumour and normal appearances of the anus
2	Excellent response with low T2 signal fibrosis only. No areas of diffusion restriction, excluding linear restriction spatially matched to anorectal mucosa
3	Indeterminate response with heterogeneous T2 signal intensity at the tumour site. Indeterminate non-linear diffusion restriction or linear restriction, not spatially matched to anorectal mucosa
4	Minimal response with reduction in size but persistent intermediate T2 tumour signal matched by restricted diffusion
5	No response of the primary tumour or frank tumour progression

### Statistical analysis

Statistical analyses were performed by a senior statistician (P.B.) using Stata (v15.1; StataCorp LP). Normally distributed variables were expressed as mean ± standard deviation. Categorical variables were expressed as absolute numbers and their percentages. The McNemar test was used to compare the number of indeterminate TRG vs. other TRG scores between reading sessions. Interobserver agreement was assessed using the kappa statistics (kappa < 0.21 = poor agreement; 0.21–0.40 = fair; 0.41–0.60 = moderate; 0.61–0.80 = good; > 0.80 = excellent). Kappa values and their standard errors were used to perform a *z*-test to compare the level of agreement between reading sessions. The Wilcoxon matched-pairs test was used to compare observer confidence scores. Analyses were performed for each observer separately, and for all observers combined. A *p* value <.05 was taken to represent statistical significance.

## Results

### Patients and clinical response

Baseline patient characteristics are summarised in Table 2. The final cohort consisted of 85 patients, ranging in age between 34 and 86 years (mean, 59 years ± 12 [SD]; 55 women). Mean tumour size was 5.1 ± 2.1 cm. A large proportion of patients had locally advanced disease at baseline (53%, 45/85), defined as T3 stage or greater, and/or tumours located in the upper canal/anorectum (52%, 44/85). Mean clinical follow-up duration was 32 ± 18 months. Clinical disease recurrence was recorded in 36% (31/85) of patients: local, in 18% (15); nodal, in 9% (8); metastatic, in 14% (12). Clinical complete tumour response was recorded in 91% (77/85) of patients at 8–10 weeks from the end of treatment. There was partial response in 6% (5/85). Progressive disease was recorded in 4% (3/85). Local recurrence was documented in 8% (7/85) of patients: 5% (4), corresponding to late recurrence, beyond 12 months from the end of treatment; 4% (3), corresponding to microscopic subclinical recurrence at 6–9 months from the end of treatment. Four patients underwent salvage surgery by means of abdominoperineal excision of the rectum.

**Table 2:** Summary of patient and tumour characteristics. Note. Values are mean ± standard deviation or number of participants with percentage of total in parentheses

Characteristic	Patients (*n* = 85)
Sex	
Female	55 (65)
Male	30 (35)
Age (year)	59 ± 12
Body mass index (kg/m^2^)	25.4 ± 5.6
TNM 8th ed. stage	
T2	40 (47)
T3	21 (25)
T4	24 (28)
N0	27 (32)
N1	58 (68)
M0	83 (98)
M1	2 (2)
Tumour size (cm)	5.1 ± 2.1
Tumour location	
Lower canal	28 (33)
Mid canal	13 (15)
Upper canal/anorectum	44 (52)
Tumour invasion of adjacent structures	
Prostate	7 (8)
Vagina	17 (20)
Ischioanal fossa	6 (7)
Tumour ADC (mm^2^/s × 10^−3^)	0.910 ± 0.182
Time interval between baseline and post treatment MRI (days)	168 ± 24
Time interval between end of treatment and post treatment MRI (days)	77 ± 17

### MRI and tumour regression grade

DWI image quality was scored as inadequate in 3 cases, which were excluded; adequate in 26% (22/85); and good in 74% (63/85). The most common problems affecting DWI quality were low signal-to-noise ratio and susceptibility artefacts, particularly from air/gas at the anal margin and in the rectal lumen. Calculated *b*-value (*b*1400 or *b*1600 s/mm^2^) images were available in 70/85 (82%) cases. Baseline tumour conspicuity on *b*800 images and ADC maps was high in all included cases, with mean tumour ADC values of 0.910 ± 0.182 × 10^−3^ mm^2^/s.

**Fig. 2:**
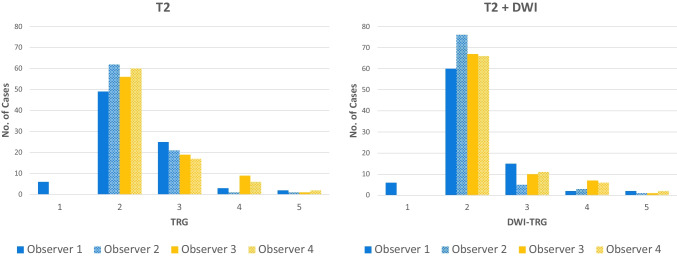
Tumour regression grade (TRG) scores amongst observers. Note. Observers 1 and 2 are expert observers. Observers 3 and 4 are non-expert observers

**Fig. 3:**
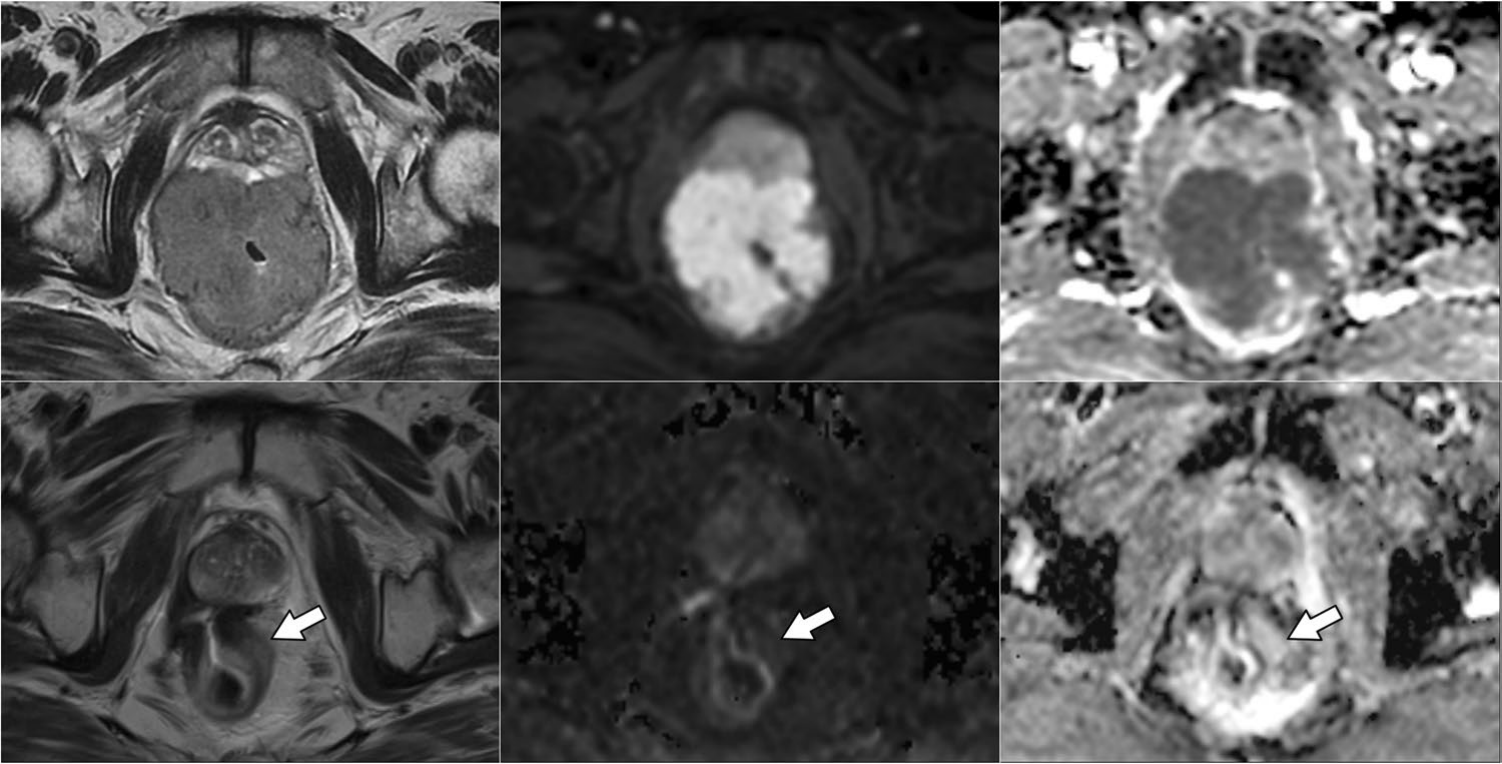
Images in a 57-year-old man with squamous anal carcinoma. T2-weighted MRI (right column), high *b*-value DWI (middle column) and DWI ADC map (left column). At baseline MRI, tumour staged as T4 invades the pelvic sidewall and prostate (upper row). After treatment (lower row), a region of indeterminate intermediate T2 signal in the lower rectal wall (TRG-3) does not correspond to restricted diffusion (arrows); linear diffusion restriction is spatially matched to anorectal mucosa (DWI-TRG-2)

**Fig. 4:**
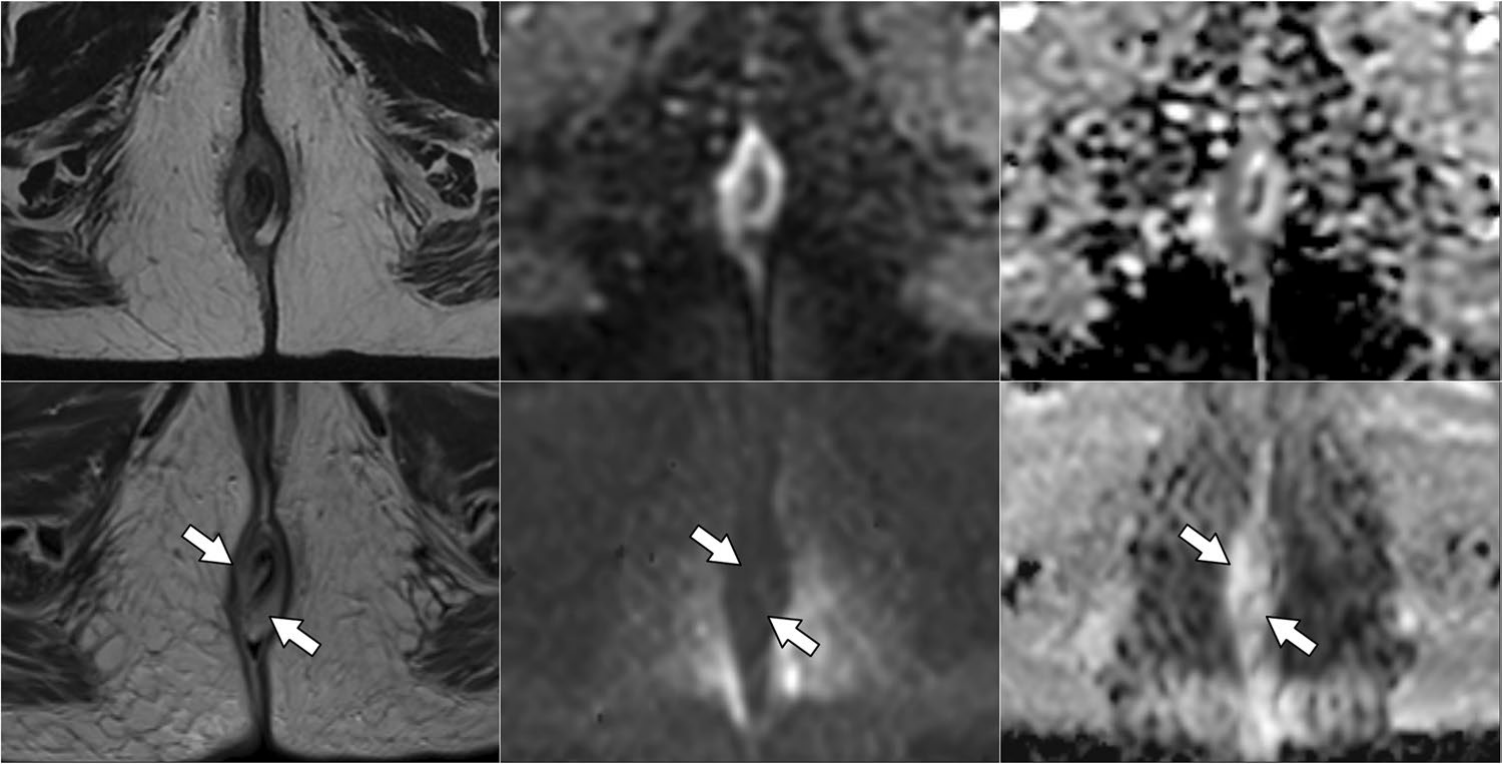
Images in a 53-year-old woman with squamous anal carcinoma. T2-weighted MRI (right column), high *b*-value DWI (middle column) and DWI ADC map (left column). At baseline MRI, tumour of the lower canal staged as T2 (upper row). Persistence of intermediate T2 signal (TRG-3) at the site of tumour (arrows) does not correspond to restricted diffusion, in keeping with excellent response (DWI-TRG-2)

**Fig. 5:**
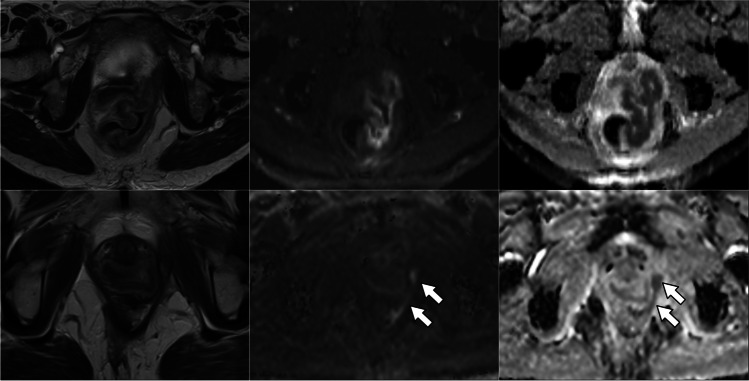
Images in a 41-year-old woman with squamous anal carcinoma. T2-weighted MRI (right column), high *b*-value DWI (middle column) and DWI ADC map (left column). At baseline MRI, tumour staged as T4 invades the vagina posteriorly and left pelvic sidewall anteriorly (upper row). After treatment (lower row), the presence of both linear and nodular diffusion restriction within a cavity left by tumour shrinkage, not spatially matched to anorectal mucosa (arrows), was deemed indeterminate (TRG-3 and DWI-TRG-3) by most observers. Complete response was recorded clinically

**Fig. 6:**
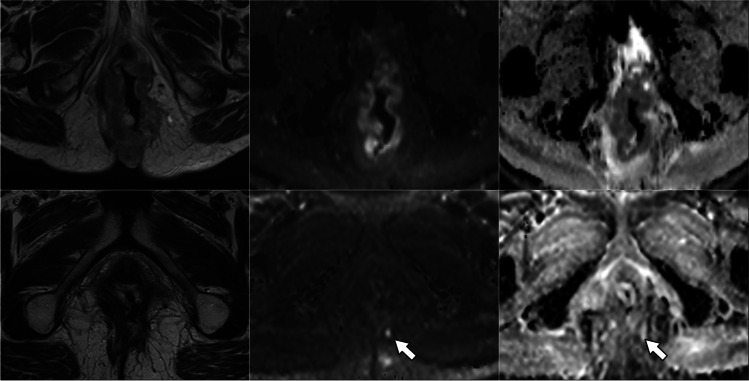
Images in a 64-year-old woman with squamous anal carcinoma. T2-weighted MRI (right column), high *b*-value DWI (middle column) and DWI ADC map (left column). At baseline MRI, tumour of the lower canal staged as T3 (upper row). After treatment (lower row), a small nodule of restricted diffusion in the 7 o’clock position (arrow) lies within a broader area of indeterminate intermediate T2 signal (TRG-3), confirming residual disease (DWI-TRG-4). Incomplete response was recorded clinically

TRG scores from the four radiologists are shown in Fig. 2. With the inclusion of DWI, the number of indeterminate TRG-3 scores decreased significantly for three of the four radiologists examined individually (difference range, 11–19%; *p* range, < 0.001–0.04), and for all radiologists combined (difference, 12%; *p* < 0.001) (Table 3). For all observers combined, the number of TRG-3 cases halved from 24% (82/340) of the total based on T2-weighted MRI alone to 12% (41/340) based on T2-weighted plus DWI. Indeterminate TRG-3 scores changed most frequently to DWI-TRG-2 (41%, 34/82), corresponding to excellent response (Figs. 3 and 4); 9% (7/82) changed to DWI-TRG-4, corresponding to minimal response (Fig. 5). The remaining 50% of TRG-3 (41/82) corresponded to indeterminate DWI-TRG-3 scores (Fig. 6).

**Table 3:** Change in the number of indeterminate tumour responses between T2-weighted MRI alone (TRG-3) and T2-weighted MRI plus DWI (DWI-TRG-3) per observer

	T2*	T2 + DWI*	Difference ^#^ (95% CI)	*p* value
Observer 1	25/85 (29%)	15/85 (18%)	−12% (−21%, −3%)	0.003
Observer 2	21/85 (25%)	5/85 (6%)	−19% (−28%, −9%)	< 0.001
Observer 3	19/85 (22%)	10/85 (12%)	−11% (−20%, −1%)	0.04
Observer 4	17/85 (20%)	11/85 (13%)	−7% (−18%, 4%)	0.16
All combined	82/340 (24%)	41/340 (12%)	−12% (−17%, −7%)	< 0.001

*Data are number of cases over total, with percentage of total in parentheses

^#^Differences calculated as value for T2+DWI minus value for T2

Observers 1 and 2 are expert observers. Observers 3 and 4 are non-expert observers

### Subjective TRG scoring confidence

Observers’ confidence in assessing response increased with the addition of DWI. Scores were higher for each of the four observers (*p* < 0.001), and for all observers combined (Supplemental Table 2). For all observers combined, 84% (287/340) of confidence scores were 4 or 5 for T2-weighted plus DWI, compared to 55% (188/340) for T2-weighted MRI alone.

### Interobserver agreement

Interobserver agreement was between fair and moderate (Supplemental Table 3), with kappa values ranging between 0.28 and 0.58. The highest agreement was achieved by non-expert observers assessing response on T2-weighted plus DWI. No significant differences in interobserver agreement were found between the two response assessment methods (*p* = 0.16–0.40).

### Correspondence between MRI TRG and clinical tumour response

Correspondence between MRI TRG scores and clinical tumour response is summarised in Table 4. Patients with complete clinical response (*n* = 77) were assigned TRG scores of 1 or 2 in 88% (270/308) of cases using T2-weighted plus DWI, versus 73% (226/308) based on T2-weighted MRI alone. Patients with partial clinical response (*n* = 5) were assigned scores of 3 or above in 75% (15/20) and 65% (13/20) of cases respectively. Eleven out of 12 TRG scores in 3 patients with progressive disease were ‘4’ or ‘5’. In 7 patients with documented local recurrence after an initial complete response, 64% (18/28) TRG-2 and 93% (26/28) DWI-TRG-2 scores were recorded, in line with complete responders without subsequent recurrence.

**Table 4:** Correspondence between MRI tumour regression grade (TRG) scores and clinical response amongst four observers

	CR (*n* = 77)		PR (*n* = 5)		PD (*n* = 3)		LRec (*n* = 7)	
TRG	T2	T2+DWI	T2	T2+DWI	T2	T2+DWI	T2	T2+DWI
1	6/308 (2)	6/308 (2)	0/20 (0)	0/20 (0)	0/12 (0)	0/12 (0)	0/28 (0)	0/28 (0)
2	220/308 (71)	264/308 (86)	7/20 (35)	5/20 (25)	0/12 (0)	0/12 (0)	18/28 (64)	26/28 (93)
3	70/308 (23)	31/308 (10)	11/20 (55)	10/20 (50)	1/12 (8)	0/12 (0)	9/28 (32)	2/28 (7)
4	12/308 (4)	7/308 (2)	2/20 (10)	5/20 (25)	5/12 (42)	6/12 (50)	1/28 (4)	0/28 (0)
5	0/308 (0)	0/308 (0)	0/20 (0)	0/20 (0)	6/12 (50)	6/12 (50)	0/28 (0)	0/28 (0)

Values are number of TRG scores with percentage of total in parentheses

*CR*, complete response; *PR*, partial response; *PD*, progressive disease; *LRec*, local recurrence

## Discussion

Assessing tumour response in the initial period following chemoradiotherapy can be challenging in patients with squamous anal carcinoma. An MRI tumour regression grade (TRG) system based on multiplanar T2-weighted sequences alone has been proposed to standardise assessment, yielding over 50% of indeterminate TRG-3 responses at 3 months from the end of treatment [10]. In our study, we found that DWI as a complement to T2-weighted sequences improved early MRI response assessment (1–3 months post treatment) by halving the total number of indeterminate responses from 24 to 12%. Qualitative evaluation of DWI signal changes, specifically the resolution of tumour hyperintensity on high *b*-value images, increased the subjective TRG scoring confidence of both expert and non-expert observers. Indeterminate TRG-3 scores changed most frequently (41%) to DWI-TRG-2, corresponding to excellent response. TRG-3 scores changed to DWI-TRG-4 in 9% of cases, indicating minimal response/residual disease, potentially allowing earlier consideration of salvage surgery, associated with favourable 5-year survival rates as high as 64% [16].

To our knowledge, external validation of the previously proposed TRG system for squamous anal carcinoma has yet to be undertaken. Our findings highlight the potential for DWI to provide early reassurance on the presence of a favourable response to definitive chemoradiotherapy, and to lower the number of patients referred for examination under anaesthesia and biopsy, given the majority were downgraded to DWI-TRG-2.

The value of DWI has already been demonstrated in rectal adenocarcinoma, where active surveillance may be considered for complete responders following neoadjuvant chemoradiotherapy [17]. DWI has higher sensitivity in restaging versus T2-weighted MRI alone (84% vs. 50%) [18]. As observed in our study, DWI improves performance by differentiating post-radiation fibrosis from viable tumour [13, 19, 20]. In rectal adenocarcinoma, active surveillance with DWI is now an alternative to surgery following neoadjuvant therapy with complete response [14].

Digital rectal examination has traditionally been the mainstay for determining complete local response in squamous anal carcinoma, and there is ongoing debate as to the benefit of imaging versus clinical evaluation. Treatment-related oedema and/or fibrosis can be difficult to distinguish from persistent active disease clinically. Treatment-related effects may even complicate the interpretation of post treatment biopsies. Proximal anorectal squamous carcinomas and locally advanced tumours represent a further challenge for clinical assessment, as their extent may not be fully appreciable by rectal examination, even under general anaesthesia [3]. It remains accepted that it may take up to 6 months for complete tumour resolution to occur. In the ACT II trial, the optimum time to assess complete response was reported as 26 weeks based on digital rectal examination and abdominopelvic CT [21]. Our findings suggest that T2-weighted MRI plus DWI may allow for earlier evaluation.

In a previous study by Kochhar et al [10], a high number of indeterminate TRG-3 scores were found in the early post treatment period (3 months post chemoradiotherapy) based on T2-weighted sequences alone, corresponding to 58% of the total. In our study, this proportion was lower, corresponding to 24% of the total based on T2-weighted sequences alone. Such difference highlights a variability in local practice, even between large-volume centres, and emphasises the need for consensus radiological guidelines for tumour response assessment.

Interobserver agreement amongst the four observers was only fair to moderate, with kappa values ranging between 0.28 and 0.58. No significant improvement in agreement was found by combining T2-weighted sequences with DWI. Importantly, non-expert observers did not show less agreement that might suggest a difficulty interpreting DWI. On the contrary, they reached the highest agreement assessing response on T2-weighted plus DWI.

A 5-point TRG system may be redundant for squamous carcinoma treated with chemoradiotherapy: only 12 patients were classified as having a TRG score of 1 or 5, as currently defined. In the study by Kochhar et al [10], no patients were scored as TRG-5 and only 2/74 patients were scored as TRG-1. A modified 3-point TRG incorporating DWI may be worth assessing in future prospective studies, as proposed for rectal cancer [22].

The value of DWI in SCCA has been investigated to date in a small number of studies, assessing its role in tumour volumetry and staging [23], and its predictive and prognostic value [24, 25]. Non-specialist or non-expert radiologists should familiarise themselves with the common interpretation pitfalls associated with DWI [26–28]. In line with previous publications, the most common problems affecting DWI quality in our study were low signal-to-noise ratio and susceptibility artefacts from air/gas at the anal margin and in the rectal lumen. All observers in our study acknowledged the value of calculated high *b*-value images in terms of T2 shine-through reduction [29].

We acknowledge several limitations. First, given the retrospective nature of our study, selection bias might have affected our results. Complex and advanced cases are referred to our tertiary surgical oncology centre. Likely because of this, a high proportion (53%) of locally advanced tumours was included in our sample. Second, due to the definitive nature of chemoradiotherapy and the high number of complete responders, it was not possible to correlate imaging with tumour histopathology after treatment in the majority of cases. Third, interpretation of post treatment MRI was particularly challenging, due to the presence of marked anatomical distortion, tumour cavities and fistulous tracts. Fourth, minor variations in the imaging acquisition across multiple 1.5- and 3.0-Tesla scanners could not be avoided. There was also some variability in the timing of post treatment MRI, which ranged between 1 and 3 months. Fifth, unlike Kochhar et al previously, we performed response MRI at a single early time point (1–3 months), instead of two time points (3 and 6 months). It is difficult to speculate how many of the TRG-3 would have resolved to TRG-2 on T2-weighted sequences alone at 6 months. Finally, our response assessment methods did not consider regional nodal response, an important prognostic factor for squamous anal carcinoma patients [3]. Combined local and regional nodal response assessment using both FDG PET-CT and MRI (including DWI) 3 months after chemoradiotherapy with curative intent was found to be the strongest predictor of patient outcome by Adusumilli et al in a single-centre series of 75 patients [30].

It must be stressed that the predictive and prognostic value of MRI response assessment against clinical reference standards remains to be proven from large prospective series in squamous anal carcinomas [9, 10]. Any conclusion regarding the predictive or prognostic value of DWI over T2-weighted sequences is beyond the scope of this study. We found no obvious correlation between MRI TRG and the onset of local recurrence after initial response.

In summary, the inclusion of DWI alongside T2-weighted MRI increases diagnostic confidence and improves early tumour response assessment in squamous anal carcinoma, by reducing the number of indeterminate responses following chemoradiotherapy.

### Supplementary information

ESM 1 (PDF 160 kb)

## Abbreviations

CR: Complete response
DWI: Diffusion-weighted imaging
LRec: Local recurrence
PD: Progressive disease
PR: Partial response
TRG: Tumour regression grade

## Declarations

### Guarantor

The scientific guarantor of this publication is Dr Davide Prezzi.

### Statistics and biometry

Mr Paul Bassett kindly provided statistical advice for this manuscript.

### Study subjects or cohorts overlap

Some study subjects (*n*=40) have been previously reported in ‘Owczarczyk K, Prezzi D, Cascino M et al (2019) MRI heterogeneity analysis for prediction of recurrence and disease free survival in anal cancer. Radiother Oncol 134:119-126’. The study’s aims differed entirely from those of this manuscript and there is no overlap of results or conclusions.

### Methodology

- retrospective
- observational
- performed at one institution
