# Correction: Diffusion-weighted imaging complements T2-weighted MRI for tumour response assessment in squamous anal carcinoma

**DOI:** 10.1007/s00330-023-10115-2

**Published:** 2023-08-28

**Authors:** Davide Prezzi, Keerthini Muthuswamy, Ashik Amlani, Kasia Owczarczyk, Ahmed Elowaidy, Tina Mistry, Paul Bassett, Vicky Goh

**Affiliations:** 1 School of Biomedical Engineering and Imaging Sciences, King’s College London, King’s Health Partners, London, UK; 2 Department of Radiology, Guy’s and St Thomas’ NHS Foundation Trust, London, UK; 3 Statsconsultancy Ltd., Amersham, UK

**Correction: European Radiology**

https://doi.org/10.1007/s00330-023-09942-0

The original version of this article, published on 18 July 2023, unfortunately contained a mistake. Reference 30 was corrected from “Lambregts DM, Rao SX, Sassen S, Martens MH et al. (2014) MRI and difusion-weighted MRI volumetry for identifcation of complete tumor responders after preoperative chemoradiotherapy in patients with rectal cancer: a bi-institutional validation study. Ann Surg 262:1034–1039”to: “Adusumilli, P., Elsayed, N., Theophanous, S. et al. Combined PET-CT and MRI for response evaluation in patients with squamous cell anal carcinoma treated with curative-intent chemoradiotherapy. Eur Radiol 32, 5086–5096 (2022). https://doi.org/10.1007/s00330-022-08648-z”. The original article has been corrected.

