# Supplementary material for: Diffusion-weighted imaging complements T2-weighted MRI for tumour response assessment in squamous anal carcinoma
Source: Eur Radiol. 2023 Jul 18;33(11):7575–84. doi: 10.1007/s00330-023-09942-0 (PMC10598114; doi:10.1007/s00330-023-09942-0)
Supplement: Supplementary file 1 — (PDF 160 kb) [file 330_2023_9942_MOESM1_ESM.pdf]

Diffusion-weighted imaging complements T2-weighted MRI for tumour response assessment in squamous anal carcinoma

Electronic Supplementary Material

Supplemental Table 1. MRI sequences and typical acquisition parameters.

	TR/TE (msec)	Slice thickness (mm)	Interslice gap (mm)	Matrix Size	FOV (cm)	No. of Signals Acquired
Sagittal T2W whole pelvis	4430/100	3	.3	307 x 384	250 x 250	2
Axial T2W whole pelvis	4590/101	5	1.5	307 x 384	300 x 300	1
Small FOV T2W perpendicular and parallel to anal canal	6530/104	3	.3	512 x 512	200 x 200	2
Axial DWI whole pelvis	5900/68	5	1.5	116 x 154	300 x 300	4

Supplemental Table 2. Observer confidence at assessing tumour response. Significant change towards high confidence scores is seen when T2-weighted MRI and DWI are used in conjunction.

	Confidence score	T2*	T2 + DWI*	*P*-Value
Observer 1	2<br>3<br>4<br>5	2 (2%)<br>31 (36%)<br>44 (52%)<br>8 (9%)	0 (0%)<br>10 (12%)<br>48 (56%)<br>27 (32%)	<0.001
Observer 2	2<br>3<br>4<br>5	14 (16%)<br>25 (29%)<br>44 (52%)<br>2 (2%)	0 (0%)<br>11 (13%)<br>30 (35%)<br>44 (52%)	<0.001
Observer 3	2<br>3<br>4<br>5	10 (12%)<br>37 (44%)<br>32 (38%)<br>6 (7%)	1 (1%)<br>17 (20%)<br>38 (45%)<br>29 (34%)	<0.001
Observer 4	2<br>3<br>4<br>5	6 (7%)<br>27 (32%)<br>38 (45%)<br>14 (16%)	1 (1%)<br>13 (15%)<br>19 (22%)<br>52 (61%)	<0.001
All combined	2<br>3<br>4<br>5	32 (9%)<br>120 (35%)<br>158 (46%)<br>30 (9%)	2 (1%)<br>51 (15%)<br>135 (40%)<br>152 (45%)	<0.001

* Data are number of cases, with percentage of total in parentheses.

Supplemental Table 3. Interobserver agreement for expert and non-expert observers for T2-weighted MRI alone and T2-weighted plus DWI.

Observers	T2 Kappa (95% CI)	T2 + DWI Kappa (95% CI)	Difference # (95% CI)	*P*-Value
Expert	0.40 (0.23, 0.56)	0.31 (0.08, 0.43)	-0.09 (-0.29, 0.12)	0.40
Non-expert	0.39 (0.23, 0.55)	0.55 (0.40, 0.71)	0.16 (-0.06, 0.38)	0.16
All	0.28 (0.22, 0.35)	0.33 (0.27, 0.40)	0.05 (-0.04, 0.14)	0.27

Difference calculated as value for T2+DWI minus value for T2
